# Grapevine mono- and sesquiterpenes: Genetics, metabolism, and ecophysiology

**DOI:** 10.3389/fpls.2023.1111392

**Published:** 2023-02-03

**Authors:** Robin Nicole Bosman, Justin Graham Lashbrooke

**Affiliations:** South African Grape and Wine Research Institute, Stellenbosch University, Stellenbosch, South Africa

**Keywords:** grapevine, terpenes, genes, metabolism, flavour, genomics, ecophysiology

## Abstract

Mono- and sesquiterpenes are volatile organic compounds which play crucial roles in human perception of table grape and wine flavour and aroma, and as such their biosynthesis has received significant attention. Here, the biosynthesis of mono- and sesquiterpenes in grapevine is reviewed, with a specific focus on the metabolic pathways which lead to formation of these compounds, and the characterised genetic variation underlying modulation of this metabolism. The bottlenecks for terpene precursor formation in the cytosol and plastid are understood to be the HMG-CoA reductase (HMGR) and 1-deoxy-D-xylylose-5-phosphate synthase (DXS) enzymes, respectively, and lead to the formation of prenyldiphosphate precursors. The functional plasticity of the terpene synthase enzymes which act on the prenyldiphosphate precursors allows for the massive variation in observed terpene product accumulation. This diversity is further enhanced in grapevine by significant duplication of genes coding for structurally diverse terpene synthases. Relatively minor nucleotide variations are sufficient to influence both product and substrate specificity of terpene synthase genes, with these variations impacting cultivar-specific aroma profiles. While the importance of these compounds in terms of grape quality is well documented, they also play several interesting roles in the grapevine’s ecophysiological interaction with its environment. Mono- and sesquiterpenes are involved in attraction of pollinators, agents of seed dispersal and herbivores, defence against fungal infection, promotion of mutualistic rhizobacteria interaction, and are elevated in conditions of high light radiation. The ever-increasing grapevine genome sequence data will potentially allow for future breeders and biotechnologists to tailor the aroma profiles of novel grapevine cultivars through exploitation of the significant genetic variation observed in terpene synthase genes.

## 1 Introduction

Terpenes, or terpenoids, are one of the most diverse classes of natural compounds with more than 80 000 identified compounds in insects, micro-organisms, and plants (Christianson, 2017). The majority of these terpenes are produced by plants where they serve various primary and secondary (or specialised) functions. Terpenes that serve vital roles in primary metabolic processes such as plant growth and development, photosynthesis, and respiration are conserved throughout the plant kingdom. These terpenes include sterols, quinones, photosynthetic pigments (chlorophylls, carotenoids), and plant hormones (brassinosteroids, abscisic acid, and gibberellins). However, in addition to these, plants produce a tremendous variety of terpenes involved in specialised metabolism, typically increasing plant fitness through their role in plant-environment interactions. So called specialised terpenes such as monoterpenes and sesquiterpenes are involved in plant-pathogen interactions, protection of plants against herbivores, and also attract pollinators and seed-dispersing animals (Dudareva et al., 2013; Vranová et al., 2013). These mono- and sesquiterpenes are characterised by their immense structural diversity which is largely due to terpene synthase (TPS) enzymes which catalyse the formation of diverse terpenes from a small pool of substrates (Degenhardt et al., 2009).

For grapevine and indeed viticulture, specialised terpenes such as, mono- and sesquiterpenes play a particularly important role in both table grape and wine aromas and are largely responsible for the distinctive flavour/aroma profile of specific cultivars. For instance, grape cultivars can be classified based on their berry monoterpene levels into three groups: muscat varieties (up to 6 mg.L^-1^ of free monoterpenes), non-muscat aromatic varieties (between 1–4 mg.L^-1^) and neutral varieties (less than 1 mg.L^-1^) (Mateo and Jiménez, 2000). While the sesquiterpene, rotundone, imparts the typical peppery aroma of Shiraz wine (Mattivi, 2016), and the monoterpene derived wine lactone leads to the sweet woody aroma of Gewurztraminer wines (Guth, 1997). Furthermore, non-volatile mono- and sesquiterpene glucosides can be enzymatically hydrolysed and released as volatiles during wine fermentation, contributing a “hidden” aromatic potential to wine (Dunlevy et al., 2009).

In grapevine, as in other plants, the first step in the biosynthesis of mono- and sesquiterpenes is the formation of prenyldiphosphate precursors, in either the cytosol (sesquiterpenes) or plastid (monoterpenes). The availability of these precursors directly regulates the capacity of the plant to synthesise volatile terpenes thereby influencing the flux of terpene metabolism. The activity of structurally diverse terpene synthases (TPSs) on the prenyldiphosphate precursors results in the diversity of terpenes produced by the plant. Additionally, these terpenes can undergo further secondary modifications, such as glycosylation and oxidation (Nagegowda & Gupta, 2020). While it has been observed that plants typically contain large *TPS* gene families, this is particularly true in grapevine, with reports of between 192-203 *TPS* genes identified in various grapevine genomes (Smit et al., 2020). While this duplication is likely due to the domestication and human selection for flavour and aroma of grapes, the eco-physiological roles of terpenes in *Vitis vinifera* are significant. Specific combinations of terpenes either attract or repel the European grapevine moth, a known grapevine pest (Salvagnin et al., 2018), while volatile terpenes induced during fungal infection and have been found to inhibit fungal growth (Simas et al., 2017; Brilli et al., 2019).

This review provides an overview of grapevine specialised terpene metabolism, focusing on monoterpene and sesquiterpene biosynthesis. The genetic and biochemical contribution of prenyldiphosphate metabolism as a regulatory point for terpene biosynthesis is highlighted, while the contribution of grapevine terpene synthases to the structural diversity of terpene compounds is discussed. Lastly, an overview of the eco-physiological functions of mono- and sesquiterpenes in grapevine is summarised.

## 2 Terpene diversity – spatial and temporal variation

Terpene profiles can vary greatly between different grapevine cultivars, as demonstrated in several studies which have characterised the terpene profile of a wide range of cultivars (Díaz-Fernández et al., 2022; D’Onofrio et al., 2017; Ji et al., 2021; Liu S. et al., 2022; Liu X. et al., 2022; Luo et al., 2019; Šikuten et al., 2022; Wu et al., 2016). However direct comparison between these studies is challenging due to differences in the methods used to quantify terpene content. Additionally, several factors influence terpene accumulation such as abiotic and biotic stress (reviewed in Rienth et al., 2021; Lazazzara et al., 2022), genetics (discussed in this review), and spatial and temporal variation (outlined here).

In grapevine, as in other plants, volatile emissions are both spatially and developmentally regulated (Abbas et al., 2017; Dudareva et al., 2013). However, unlike several other plants, grapevine does not accumulate terpenes and other volatiles in specialised organs. Generally, monoterpenes are most abundant in the berry skin, with some monoterpenes being present in the berry pulp (Wu et al., 2016; Lin et al., 2019). Sesquiterpenes are most abundant in grapevine flowers and in early fruit development (Martin et al., 2009; Matarese et al., 2014; Smit et al., 2019). A study by Matarese et al. (2014) analysed the VOCs present in various grapevine organs and found a clear distinction between the terpene profiles present in different organs with roots having the most distinctive volatile profile and grapevine flowers found to have the highest volatile terpene content. These results indicate the specialisation of terpenes in different grapevine organs, which is likely due to evolved ecophysiological roles of specialised terpenes and human selection. In grapevine, the accumulation of terpenes over development has been mostly limited to grape berries and specifically focused on monoterpenes. Generally, monoterpene content is found to increase over the course of berry development (Ji et al., 2021; Liu X. et al., 2022; Luo et al., 2019; Martin et al., 2012). Research on the evolution of sesquiterpenes over development is limited due to their low levels of accumulation in grape berries (Dunlevy et al., 2009; Lin et al., 2019).

## 3 Biosynthesis of the prenyldiphosphate precursors of terpenes

### 3.1 Key enzymes of the MVA and MEP pathways

**Figure 1:**
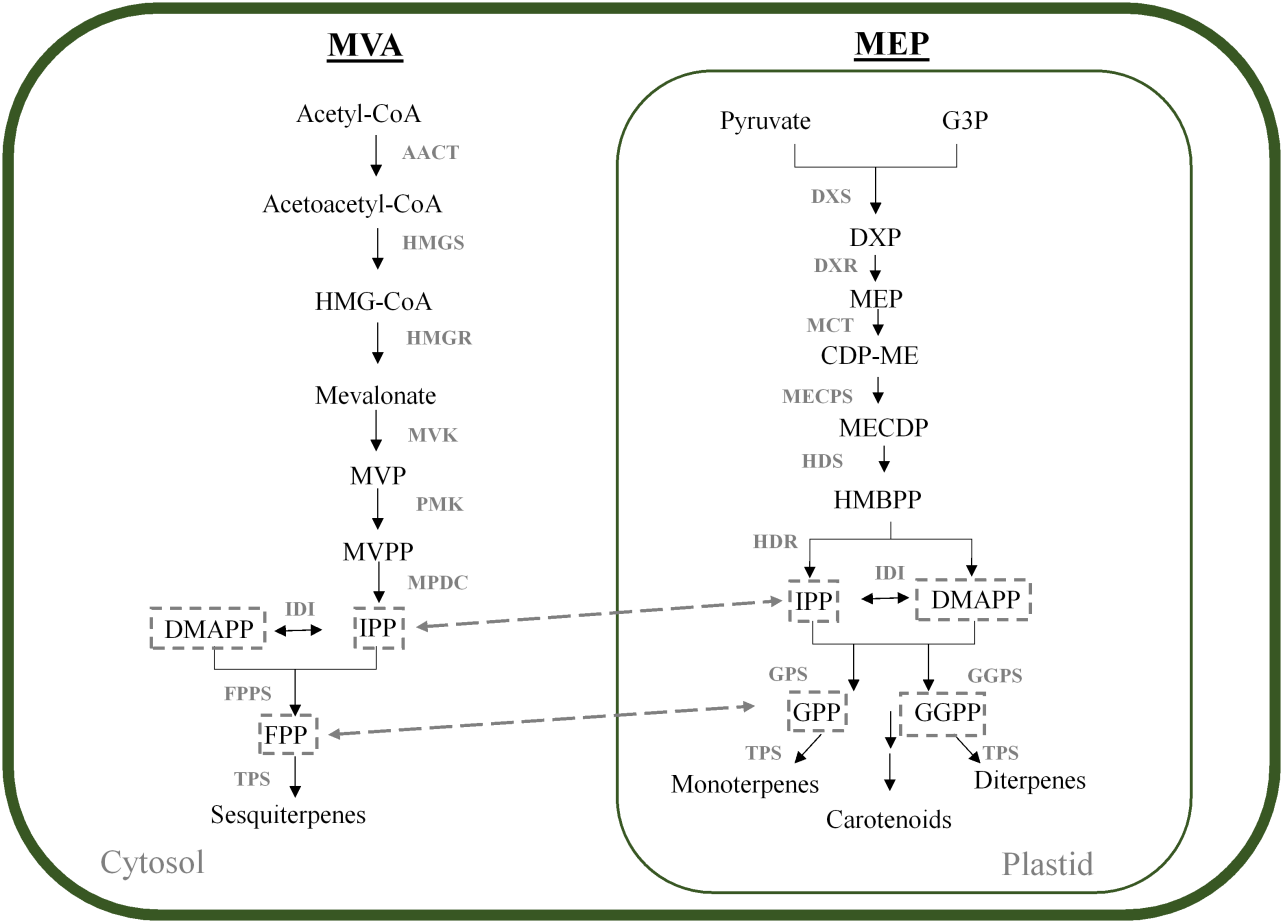
MVA and MEP pathways in plants. The MVA and MEP pathways forming the prenyldiphosphate precursor molecules for terpene synthesis are shown in the cytosol and plastid, respectively. Downstream metabolites are indicated. AACT, acetyl-CoA acetyltransferase; CDP-ME, 4-diphosphocytidyl-2-C-methyl-D-erythritol; CDP-MEP, CDPME 2-phosphate; CMK, 4-(cytidine 5′-diphospho)-2-C-methyl-D-erythritol kinase; DMAPP, dimethyl allyl diphosphate; DXP, 1-deoxy-D-xylulose 5-phosphate; DXS, DXP synthase; DXR, 1-deoxy-D-xylulose 5-phosphate reductoisomerase; FPP, farnesyl diphosphate; FPPS, FPP synthase; G3P, glyceraldehyde 3-phosphate; GGPP, geranylgeranyl diphosphate; GGPPS, GGPP synthase; GPP, geranyl diphosphate; GPPS, GPP synthase; HDR, hydroxymethylbutenyl diphosphate reductase; HDS, 4-hydroxy-3-methylbut-2-en-1-yl diphosphate synthase; HMBPP, (*E*)-4-hydroxy-3-methylbut-2-en-1-yl diphosphate; HMG-CoA, hydroxymethylglutaryl-CoA; HMGR, HMG-CoA reductase; HMGS, HMG-CoA synthase; IDI, isopentenyl pyrophosphate isomerase; IPP, isopentenyl diphosphate; MCT, 2-C-methyl-D-erythritol 4-phosphate cytidylyltransferase; MECPD, 2-C-methyl-D-erythritol 2,4-cyclodiphosphate; MECPS, MECPD synthase; MVK, mevalonate kinase; MPDC, mevalonate diphosphate decarboxylase; MVP, mevalonate 5-phosphate; MVPP, mevalonate 5- pyrophosphate; PMK, phosphomevalonate kinase; TPS, terpene synthase.

Monoterpenes and sesquiterpenes, like all other terpenes, are derived from the C_5_ isoprene precursors isopentenyl diphosphate (IPP) and dimethyl allyl diphosphate (DMAPP) (Tholl, 2015). Plants employ two independent pathways to produce these precursors, namely the mevalonate pathway (MVA) and the methylerythritol phosphate (MEP) pathway which are compartmentalised into the cytoplasm and plastids, respectively (**Figure 1**). Compartmentalisation of MVA and MEP intermediates is not strict, and it has been shown that intermediates can be exchanged across the plastidial membrane in a process termed “metabolic crosstalk” (Gutensohn et al., 2013; Schwab and Wüst, 2015). Metabolites which are exchanged between these pathways include IPP itself, as well as the prenyldiphosphate precursors of terpene biosynthesis, geranyl diphosphate (GPP), farnesyl diphosphate (FPP) and geranylgeranyl diphosphate (GGPP) (reviewed in Hemmerlin et al., 2012; Gutensohn et al., 2013; Liao et al., 2016). **Table 1** shows enzymes of the MVA and MEP pathway which have been characterised in grapevine.

**Table 1:** Functionally characterised grapevine genes involved in terpene biosynthesis.

Gene name	Closest PN40024 v3 gene model	Major product(s)	Substrate(s)	Cultivar	Type of characterisation study (eg. *in vitro, in planta*)	Reference
MVA and MEP pathway enzymes						
*VvDXS1*	N/A	1-deoxy-D-xylulose 5-phosphate	Pyruvate and glyceraldehyde 3-phosphate	Moscato Bianco	Enzyme assay and heterologous *in planta* expression	(Battilana et al., 2011)
*VvHMGR3*	N/A	Mevalonate	HMG-CoA	Kyoho	Transient heterologous *in planta* expression	(Zheng et al., 2020)
TPS-a subfamily						
*VvGwECar1*	TPS03	(*E*)-caryophyllene	FPP	Gewürztraminer	*In vitro* enzyme assay	(Martin et al., 2010)
*VvGwECar2*	TPS27	(*E*)-caryophyllene	FPP	Gewürztraminer	*In vitro* enzyme assay	
*VvGwECar3*	TPS02	(*E*)-caryophyllene	FPP	Gewürztraminer	*In vitro* enzyme assay	
*VvPNECar1*	TPS02	(*E*)-caryophyllene	FPP	Pinot Noir	*In vivo* enzyme assay	
*VvPNECar2*	TPS13	(*E*)-caryophyllene	FPP	Pinot Noir	*In vivo* enzyme assay	
*VvGwGerA*	TPS03	Germacrene A	FPP	Gewürztraminer	*In vitro* enzyme assay	
*VvGwaBer*	TPS10	(*E*)-α-bergamotene	FPP	Gewürztraminer	*In vitro* enzyme assay	
*VvGwGerD*	TPS07	germacrene D	FPP	Gewürztraminer	*In vitro* enzyme assay	
*VvPNGerD*	TPS15	germacrene D	FPP	Pinot Noir	*In vitro* enzyme assay	
*VvCSaFar*	TPS20	(*E,E*)-α-Farnesene	FPP	Cabernet Sauvignon	*In vitro* enzyme assay	
*VvGwgCad*	TPS08	γ-Cadinene	FPP	Gewürztraminer	*In vitro* enzyme assay	
*VvPNbCur*	TPS30	β-curcumene	FPP	Pinot Noir	*In vitro* enzyme assay	
*VvPNSesq*	TPS12	sesquithujene	FPP	Pinot Noir	*In vivo* enzyme assay	
*VvPNaZin*	TPS14	α-zingiberene	FPP	Pinot Noir	*In vivo* enzyme assay	
*VvPNSeInt*	TPS24	selina-4,11-diene	FPP	Pinot Noir	*In vivo* enzyme assay	
*VvPNCuCad*	TPS26	Cubebol δ-Cadinene	FPP	Pinot Noir	*In vivo* enzyme assay	
*VvPNaHum*	TPS11	α-humulene	FPP	Pinot Noir	*In vivo* enzyme assay	
*VvPNEb2epi Car*	TPS21	(E)-β-caryophyllene 2-epi-(*E*)-β-Caryophyllene	FPP	Pinot Noir	*In vivo* enzyme assay	
*VvGuaS*	TPS24	α-guaiene	FPP	Shiraz	Heterologous *in planta* expression	(Drew et al., 2016)
*VvGerD*	TPS28	Germacrene D	FPP	Gewürztraminer	*In vitro* enzyme assay	(Lücker et al., 2004)
*VvVal*	TPS15	(+)-valencene	FPP	Gewürztraminer	*In vitro* enzyme assay	
*VvSBTPS01*	TPS01	α-Selinene		Sauvignon Blanc	*In vivo* enzyme assay	(Smit et al., 2019)
*VvMATPS01*	TPS01	α-Selinene		Muscat of Alexandria	*In vivo* enzyme assay	
*VvSBTPS02*	TPS02	(*E*)-β-Caryophyllene		Sauvignon Blanc	*In vivo* enzyme assay	
*VvMATPS10*	TPS10	(*E*)-β-Farnesene		Muscat of Alexandria	*In vivo* enzyme assay and Heterologous *in planta* expression	
*VvSHTPS27*	TPS27	(*E*)-β-Caryophyllene		Shiraz	*In vivo* enzyme assay	
*VvMATPS27*	TPS27	(*E*)-β-Caryophyllene		Muscat of Alexandria	*In vivo* enzyme assay	
*VvShirazTPS07*	TPS07	Ylangene Germacrene D		Shiraz	Heterologous *in planta* expression	(Dueholm et al., 2019)
*VvShirazTPS26*	TPS26	α-Cubebene α-Copaene δ-Cadinene		Shiraz	Heterologous *in planta* expression	
*VvShirazTPS27*	TPS27	Isocaryophyllene		Shiraz	Heterologous *in planta* expression	
*VvShirazTPS-Y1*	TPS28	δ-Cadinene		Shiraz	Heterologous *in planta* expression	
*VvShirazTPS-Y2*	TPS29	Isocaryophyllene β-cadinene		Shiraz	Heterologous *in planta* expression	
TPS-b subfamily						
*VvTer*	TPS39	α-terpineol	GPP	Gewürztraminer	*In vitro* enzyme assay	(Martin & Bohlmann, 2004)
*VvGwaPhe*	TPS45	(+)-α-phellandrene	GPP	Gewürztraminer	*In vitro* enzyme assay	(Martin et al., 2010)
*VvPNaPin1*	TPS44	(+)-α-pinene	GPP	Pinot Noir		
*VvPNaPin2*	TPS44	(+)-α-pinene	GPP	Pinot Noir		
*VvGwbOci*	TPS34	(*E*)-β-ocimene	GPP	Gewürztraminer		
*VvCSbOci*	TPS35	(*E*)-β-ocimene	GPP	Cabernet Sauvignon		
*VvCSbOciM*	TPS39	(*E*)-β-Ocimene/Myrcene	GPP	Cabernet Sauvignon		
*VvGwbOciF*	TPS46	(*E*)-β-Ocimene *(E,E*)-α-Farnesene	GPP FPP	Gewürztraminer		
*VvPNRLin*	TPS31	(*3R*)-Linalool	GPP	Pinot Noir		
TPS-g subfamily						
*VvGwGer*	TPS52	Geraniol	GPP	Gewürztraminer	*In vitro* enzyme assay	(Martin et al., 2010)
*VvCSGer*	TPS51	Geraniol	GPP	Cabernet Sauvignon		
*VvPNGer*	TPS52	Geraniol	GPP	Pinot Noir		
*VvPNLinNer1*	TPS59	(*3S*)-Linalool (*E*)- Nerolidol	GPP FPP	Pinot Noir		
*VvPNLinNer2*	TPS56			Pinot Noir		
*VvCSLinNer*	TPS56			Cabernet Sauvignon		
*VvPNLNGl1*	TPS57	Linalool *(E*)- Nerolidol (*E,E*)-Geranyl-linalool	GPP FPP GGPP	Pinot Noir		
*VvPNLNGl2*	TPS63			Pinot Noir		
*VvPNLNGl3*	TPS53			Pinot Noir		
*VvPNLNGl4*	TPS53			Pinot Noir		
Terpene modifying enzymes						
*VvSTO2*	N/A	Rotundone	α-guaiene	Syrah	*In vitro* enzyme assay	(Takase et al., 2016a)
*VvCYP76F14*	N/A	(*E*)-8-carboxylinalool	Linalool	Gewurztraminer	*In vitro* enzyme assay and Transient heterologous *in planta* expression	(Ilc et al., 2017)
*VvGT7*	N/A	geranyl and neryl glucoside	geraniol, nerol, and citronellol	Gewurztraminer and White Riesling	*In vitro* enzyme assay	(Bönisch et al., 2014a)
*VvGT14*	N/A	geranyl and neryl glucoside	geraniol, nerol, citronellol and linalool	Gewurztraminer and White Riesling	*In vitro* enzyme assay	(Bönisch et al., 2014b)
*VvGT15*	N/A	geranyl and neryl glucoside	geraniol, nerol, and citronellol	Gewurztraminer and White Riesling	*In vitro* enzyme assay	(Bönisch et al., 2014b)

The first step in the MEP pathway is catalysed by 1-deoxy-D-xylylose-5-phosphate synthase (DXS), an enzyme which plays a major contribution to metabolic flux control in plastidial terpene biosynthesis (Tholl, 2015). Grapevine DXS (VvDXS1) has been established as an important contributor to the aroma of Muscat cultivars (Doligez et al., 2006; Battilana et al., 2009). Battilana et al. (2009) reported that *VvDXS1* co-localizes with a major QTL on linkage group 5 which associates with three monoterpenes: linalool, nerol, and geraniol, which are responsible for the distinct floral and citrus aromas of Muscat cultivars. Further studies of *VvDXS1* found a single nucleotide polymorphism (SNP) at position 1822 (G substituting a T) that was hypothesised to be a “gain of function” mutation (Emanuelli et al., 2010). *VvDXS1* genes that were heterozygous (GT) at position 1822 caused a non-synonymous substitution of a lysine (K) with an asparagine (N) at position 284 of the VvDXS1 protein. Functional characterisation of VvDXS1 showed that the non-synonymous amino acid substitution influences enzyme kinetics by increasing the catalytic efficiency of VvDXS1, thereby increasing the total monoterpene content of cultivars carrying this SNP (Battilana et al., 2011). This was further supported by transgenic tobacco lines overexpressing the K284N SNP allele of *VvDXS1* showing up to 20 times higher levels of glycosylated monoterpenes than lines expressing the neutral allele (Battilana et al., 2011). Additionally, microvine lines overexpressing the neutral and muscat allele of *VvDXS1* had a 1.7- and 4.4-fold increase in total monoterpene content compared to the wild type, respectively (Dalla Costa et al., 2018). The K284N SNP of VvDXS1 appears to be a reliable marker for muscat-aroma in grapevine cultivars. A recent study looking at the association between *VvDXS1* and aromatic substance content in different flavour types (muscat-like, aromatic and neutral aroma) of grapevine varieties also associated the K284N SNP with increased monoterpene content in grapevine (Yang et al., 2017b).

Dalla Costa et al. (2018) investigated the effect of the K284N SNP on terpene content in 90 grapevine germplasms. Predictably, cultivars that were homozygous (TT) or heterozygous (GT) for the Muscat-allele had a significantly higher level of monoterpenes than cultivars homozygous (GG) for the neutral allele. Interestingly, the authors also reported a similar trend, albeit to a lesser extent, in sesquiterpene content. Furthermore, overexpression of *VvDXS1* in combination with *VvLinNer* (see **Table 1**), a linalool/nerolidol synthase, led to a significant increase in linalool (a monoterpene) and nerolidol (a sesquiterpene) content (Wang et al., 2021). The increase in sesquiterpene content associated with *VvDXS1* overexpression may be explained by the phenomenon of “metabolic cross-talk” between the MEP and MVA pathways. The increased flux towards plastid-bound MEP pathway precursors, due to overexpression of *VvDXS1*, potentially leads to an increase in transport of these precursors to the cytosol where they are incorporated in the MVA pathway resulting in an increase in sesquiterpene biosynthesis.

While *VvDXS1* is an effective marker for muscat-aroma, it is not the sole determinant of monoterpene biosynthesis. Emanuelli et al. (2010) found that several cultivars which are characterised as aromatic show no presence of the K284N SNP. Indeed, out of 20 aromatic cultivars, it was reported that 75% are homozygous for the neutral allele (Emanuelli et al., 2010). Therefore, the monoterpene content of aromatic cultivars is likely influenced by enzymes other than VvDXS1. Furthermore, three cultivars with muscat-like aroma but no Muscat parentage were also shown to be homozygous for the neutral allele. However, these three cultivars (Gewürztraminer, Chardonnay musqué clone 44-60 Dijon, and Chasselas musqué) each had unique heterozygous SNPs in *VvDXS1* located close to the K284N SNP. Further investigation of these SNPs is necessary to determine whether they are associated with increased monoterpene accumulation in Muscat-like aromatic cultivars.

While apparently predominantly controlled by DXS, the metabolic flux through the MEP pathway in plants is further regulated by other enzymes which include hydroxymethylbutenyl diphosphate reductase, HDR (Vranová et al., 2013). In grapevine, the expression of *VvHDR* has been shown to correlate with the veraison-initiated accumulation of monoterpenes in certain cultivars (Martin et al., 2012; Wen et al., 2015; Costantini et al., 2017; Yue et al., 2020), indicating the potentially regulatory role of *VvHDR* in grapevine monoterpene biosynthesis.

A major contributor to metabolic flux control of the MVA pathway is HMG-CoA reductase (HMGR) (Rodríguez-Concepción, 2006). Three HMGRs have been identified in grapevine, *VvHMGR1-3* (Zheng et al., 2020; Zheng et al., 2021). The three genes are differentially expressed in grapevine organs and during berry development. Interestingly, it was found that *VvHMGR3* plays a role in fruit colour formation. Heterologous suppression of *VvHMGR3* in strawberry increased the rate of colour formation and increased anthocyanin formation and inversely, overexpression of *VvHMGR3* suppressed colour (Zheng et al., 2020). Furthermore, the authors found that brassinosteroids (BRs) (which are produced *via* the MVA pathway) inhibit *VvHMGR* expression. A BR-HMGR model is proposed whereby *VvHMGR* expression leads to an increase in BR accumulation and in turn BRs have negative feedback on HMGR activity. Additionally, BRs increases anthocyanin content.

### 3.2 GGP, FPP and GGPP

The final products of the MEP and MVA pathways, IPP and DMAPP, are fused through consecutive head-to tail condensation reactions, catalysed by short chain prenyltransferases, to form prenyl diphosphates which serve as the precursor backbones for terpenoids (Vranová et al., 2013; Tholl, 2015).

C_10_ Geranyl diphosphate (GPP) is the precursor for monoterpene biosynthesis and is formed through the activity of GPP synthases (GPPS). Plant GPPSs exist as either hetero- or homodimeric enzymes (Nagegowda & Gupta, 2020). Heterodimeric GPPS consists of a large subunit (LSU) and a catalytically inactive small subunit (SSU-I). GPPS-LSU shares high homology with geranylgeranyl diphosphate synthase (GGPPS) and in some instances has been shown to possess GGPPS activity as a homodimer (Tholl, 2015). GGPP is the precursor molecule to many other primary and specialised terpenes such as carotenoids, abscisic acid, chlorophylls, phytol tocopherols, gibberellins, plastoquinones, polyprenols, and diterpenoids.

To date, very little research has been done on grapevine GPPSs (VvGPPSs). Early reports indicate that VvGPPSs are localised to the plastids, as is the case with plant GPPSs in general (Feron et al., 1990; Soler et al., 1992). More recently, gene expression and transcriptomic studies investigated the expression of *VvGPPS* with respect to terpene accumulation. Transcript abundance levels of the *VvGPPS* gene were shown to parallel the veraison-initiated accumulation of monoterpenes and is potentially contributes to flux control in monoterpene biosynthesis (Martin et al., 2012; Wang et al., 2021). However, these studies do not differentiate between GPPS-LSU and GPPS-SSU. An integrated transcriptomic and metabolomic study of ripening Moscato Bianco berries showed no correlation between *VvGPPS-LSU* and terpene accumulation (Costantini et al., 2017).

C_15_ farnesyl diphosphate (FPP), produced by FPP synthase (FPPS) is the precursor for sesquiterpenes, triterpenes and primary metabolites such as phytosterols, brassinosteroids, dolichols and ubiquinones (Tholl, 2015). Plant FPPSs are homodimeric enzymes and have been reported to localise to cytosol, mitochondria, or peroxisomes in different plant species (Tholl, 2015). As with VvGPPSs, the molecular functional characterisation of grapevine FPPSs (VvFPPSs) is limited. The importance of *VvFPPS* in grapevine sesquiterpene biosynthesis is highlighted in a study analysing the transcription of genes related to the biosynthesis of rotundone, the sesquiterpene responsible for the peppery aroma of Syrah cultivars. This work indicated that *VvFPPS* potentially plays a vital role in the accumulation level of rotundone in Syrah cultivars by increasing the substrate pool available for rotundone precursor synthesis (Takase et al., 2016a).

### 3.3 Alternative routes for terpene biosynthesis

Plant GPPSs and FPPSs were generally accepted to be *trans* prenyltransferases, i.e., they synthesise the trans (*E*) conformation of GPP and FPP, respectively, however, short-chain *cis* prenyltransferases have been identified in several plants (Akhtar et al., 2013; Demissie et al., 2013). For example, a *cis* FPPS (zFPPS) was demonstrated to produce *Z,Z*-FPP in the glandular trichomes of tomatoes (Sallaud et al., 2009). zFPPS is localised to the plastids, unlike cytosolic *trans* FPPS, and is therefore theorised to use precursors from the MEP pathway. Furthermore, *Z,Z*-FPP was shown to be used as a substrate for sesquiterpene synthase. To date, tomato is the only species where *cis* prenyldiphosphates have been reported as terpene synthase (TPS) substrates.

Another non “traditional” route for terpene biosynthesis was shown through the function of isopentenyl phosphate kinases (IPKs). IPS catalyses the conversion of isopentenyl phosphate (IP) and possibly dimethylallyl phosphate (DMAP) to IPP and DMAPP, respectively (Henry et al., 2018). The presence of genes encoding IPKs in all sequenced plant genomes indicate a possible regulatory role for terpene biosynthesis *via* IPK *via* IPP/IP and DMAPP/DMAP ratio modulation (Nagegowda & Gupta, 2020). IP and DMAP was shown to be produced through dephosphorylation of IPP (and DMAPP) by members of the Nudix hydrolase super family (AtNudx1 and AtNudx3) (Henry et al., 2018). Nudix hydrolyses in rose has also recently been reported to provide a TPS-independent path for monoterpene production (Magnard et al., 2015). Rose Nudix hydrolase (RhNudx1) catalyses the formation of geranyl monophosphate (GP) from GPP; GP is then further converted to geraniol by an unidentified phosphatase. With regards to grapevine, a nudix hydrolase (VIT_10s0003g00880), whose expression increased along berry development and correlated with linalool content in Moscato Bianco was proposed as a candidate gene for an alternative route of monoterpene production in grapevine (Costantini et al., 2017).

## 4 Terpene synthases

The major contributor to the diversity of terpenes are terpene synthases (TPSs) which catalyse the formation of terpenes from prenyldiphosphate precursors, e.g. GPP, FPP and GGPP. The ability of TPSs to produce this wide variety is due to various structural features of the enzyme, as well as rapid evolutionary diversification. The following sections explore the contribution of TPSs to grapevine terpene diversity.

### 4.1 Plant terpene synthase gene family

**Figure 2:**
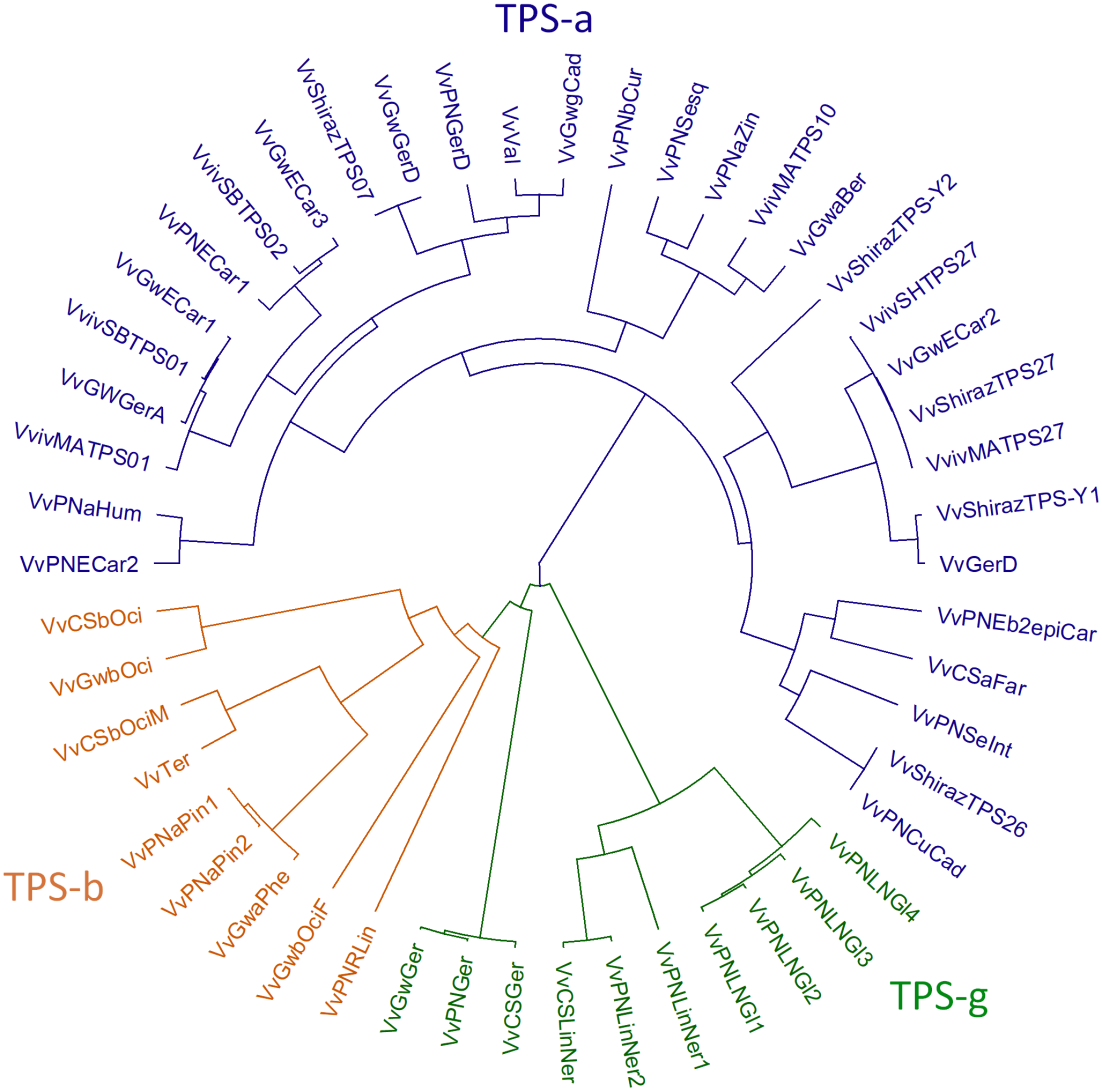
Phylogenetic tree of characterised grapevine terpene synthases. The TPS-subfamilies a, b and g are indicated in blue, orange and green, respectively.

The plant TPS gene family is mid-sized with TPS genes typically ranging from 30-170 per plant species. The gene family has been divided into seven subfamilies (TPS-a, -b, -c, -d, -e/f, -g and -h) based on their sequence similarity and proposed function (Bohlmann et al., 1998; Chen et al., 2011). The TPS-c and TPS-e/f subfamilies are involved in primary metabolism, encoding copalyl diphosphate synthases (CPSs) and kaurene synthase (KSs) which are involved in gibberellin biosynthesis (Chen et al., 2011). The TPS-d family produce gymnosperm-specific terpene synthases, while TPS-h genes encode putative bifunctional diterpene synthases in the lycophyte *Selaginella moellendorffii* (Chen et al., 2011). TPS-a, b, and g families are angiosperm-specific and produce mono-, sesqui-, and diterpenes involved predominantly in specialised metabolism (Chen et al., 2011). TPS-a subfamily members typically produce sesquiterpenes and diterpenes, while members of the TPS-b and TPS-g subfamilies produce monoterpenes, although the TPSs from the TPS-g subfamily exclusively produce acyclic terpene alcohols. Grapevine TPSs (*VvTPS*s) fall within every subfamily except TPS-d and TPS-h (Martin et al., 2010; Smit et al., 2020). More than half of the specialised *VvTPSs* are sesquiterpene synthases (TPS-a), with the rest making up the TPS-b and -g subfamilies. Several *VvTPS*s from a number of cultivars have been functionally characterised and are summarised in **Table 1**. Additionally, **Figure 2** shows a phylogenetic tree of functionally characterised VvTPSs.

The expansion of the TPS gene family within plants is thought to occur primarily through tandem or segmental gene duplication. The highly inbred and homozygous Pinot Noir genome has served as the reference genome (PN40024) for grapevine for the last decade (Jaillon et al., 2007). In a keystone study for grapevine TPSs by (Martin et al., 2010), using the reference genome, the grapevine TPS gene family was functionally annotated which revealed that this gene family is substantial with 69 putatively functional TPSs. The increasing number of available genome sequences for different grapevine cultivars shows that due to its homozygosity PN40024 is limiting to our understanding of *VvTPS*s. Indeed, a comparison of the draft diploid genomes of Cabernet Sauvignon, Carménère, and Chardonnay to PN40024 revealed that there is a larger number of *VvTPS*s in these cultivars than the canonically accepted 69 (Smit et al., 2020). Furthermore, the number of *VvTPS*s varies significantly between cultivars, ranging from approximately 80 to 200. Grapevine genomes show extensive duplication events, which led to the expansion of the *VvTPS* family and the fact that 69-90% of *VvTPSs* in grapevine are related to gene duplication events (Jiang et al., 2019; Smit et al., 2020). Additionally, annotation of *VvTPS*s revealed that the majority of sesquiterpene synthases cluster on chromosome 18, while monoterpene synthases cluster on chromosome 13 (Martin et al., 2010; Smit et al., 2020). Another interesting feature of *VvTPS*s revealed by the sequencing of diploid genomes is that approximately 30% of *VvTPS*s are hemizygous, which can play a vital role in understanding the inheritance of these genes for molecular breeding. Taken together, these factors including the large gene family size, cultivar variation, extensive duplication, and hemizygosity of *VvTPS*s goes a long way in explaining the large variation in terpene composition observed in different grapevine cultivars and highlights the immense potential of grapevines to produce novel and diverse terpenes.

Extensive gene duplication of TPSs has also been associated with the variation in plant terpene composition in different organs and at different developmental stages. Duplicated TPS genes serve the same enzymatic function, but often show divergent temporal and spatial expression patterns resulting in tissue or time specific terpene profiles. These sub-functionalisation events have also been demonstrated in grapevine. Expression analysis of gene paralogs of (*E*)-β-caryophyllene synthases, β-ocimene synthases, and linalool synthases showed that all gene paralogs were differentially expressed during Moscato Bianco grape berry development (Matarese et al., 2013). Beyond the grape berry, these gene paralogs were also differentially expressed in different grapevine organs (Matarese et al., 2013; Matarese et al., 2014). Differential gene expression patterns for paralogs of β-ocimene synthases and linalool synthases were also shown in Sauvignon Blanc, Riesling, and Hamburg Muscat berry development (Yue et al., 2020). Moreover, the expression pattern of gene paralogs differed between cultivars, further showcasing the high level of variation of *VvTPS*s and terpene accumulation between grapevine cultivars.

### 4.2 Terpene synthases: enzyme structure and function

Terpene synthase enzymes can be divided into two classes (type I TPSs or type II TPSs) based on their mechanism of catalysis. Class II TPSs catalyse the ionisation of GGPP *via* protonation. More common are the class I TPSs which contain all mono- and sesquiterpenes. Class I TPSs catalyse the ionisation of the phosphate group on the prenyldiphosphate substrate (GPP, FPP, or GGPP), forming a highly reactive carbocation intermediate which can undergo various reactions such cyclisations or hydride shifts until the reaction ends with proton loss or the addition of a nucleophile (Degenhardt et al., 2009).

TPSs have various protein motifs that play an important role in their enzyme function. Class I TPSs contains two aspartate-rich motifs, DDxxD and NSE/DTE in the C-terminal domain which flank the active site. DDxxD and NSE/DTE both bind a trinuclear magnesium cluster which is involved in the positioning of the substrate (Degenhardt et al., 2009). Unlike the DDxxD motif, which is highly conserved through all plant TPSs, the NSE/DTE motif is less conserved with a consensus sequence of (L,V)(V,L,A)-(N,D)D(L,I,V)x(S,T)xxxE (Degenhardt et al., 2009). Upstream of the DDxxD motif is a highly conserved RxR motif which prevents nucleophilic attack on any of the carbocationic intermediates (Degenhardt et al., 2009). An altered RxQ motif appears in sesquiterpene synthases which produce nerolidol, an acyclic terpene. This altered motif may be less effective at preventing nucleophilic attack of the carbocationic intermediate leading to the termination of the enzyme reaction before cyclisation can occur (Durairaj et al., 2019). On the N-terminal end, TPSs contain an RRx_8_W motif which has been predicted to play a role in terpene cyclization. Mono- and diterpene synthases contain an N-terminal plastid transit peptide upstream of the RRx8W motif resulting in their localization to plasmids.

In the grapevine TPS-g subfamily the RRx8W motif is not well conserved (Martin et al., 2010) which supports the proposed involvement of the RRx8W motif in cyclisation as TPSs from the TPS-g subfamily primarily produce acyclic monoterpene alcohols. Interestingly, the NSE/DTE motif of the TPS-g subfamily in grapevine has a modified and highly conserved sequence LWDDLx(S,T)xxxE (Martin et al., 2010). The NSE/DTE motif may thus play a role in determining the cyclisation function in grapevine TPSs.

### 4.3 Substrate and product specificity of TPSs

In the plant kingdom, several multi-substrate TPSs which can use GPP, FPP, and GGPP *in vitro* to produce monoterpenes, sesquiterpenes, and diterpenes respectively, have been identified (reviewed in (Pazouki and Niinemetst, 2016). Three multi-substrate TPSs have been characterized in grapevine, namely, VvPNLinNer1, VvPNLinNer2, and VvCSLinNer, capable of producing linalool (a monoterpene) and nerolidol (a sesquiterpene) from GPP and FPP, respectively (**Table 1**) (Martin et al., 2010). Additionally, four TPSs, VvPNLNGl1-4, also accepted GGPP to produce (*E,E)*-geranyl linalool (Martin et al., 2010). VvGwbOciF and VvCSbOciF could also accept both GPP and FPP to produce (*E*)-β-ocimene or (*E,E*)-α-farnesene, respectively. Lastly, VvCSENerGl and VvPNENerGl accepted either FPP or GGPP to produce *E*-nerolidol or (*E,E)*-geranyl linalool, respectively (Martin et al., 2010). These enzymes were characterised *in vivo* using metabolically engineered *E. coli.* Subcellular localisation of Riesling VvLinNer (VvRiLinNer) showed that the enzyme is localized to the chloroplasts and the authors proposed that due to its localisation, VvRiLinNer could only produce linalool *in planta* (Zhu et al., 2014). This inference is supported by a previous study that demonstrated grape derived monoterpenes are almost exclusively synthesised *via* the plastid-localised MEP pathway (Luan, 2002), while cytosolic localised sesquiterpenes are produced from both the cytosolic MVA and plastidial MEP pathway intermediates (May et al., 2013). Contrarily, a recent study reported that *N. benthimiana* leaves transiently overexpressing *VvLinNer* (isolated from the cultivar Shine Muscat) had elevated levels of both linalool and nerolidol, with linalool being predominant (Wang et al., 2021). Seeing as VvLinNer is localised to the plastids, this recent finding potentially demonstrates that the substrate pool in plastids may be derived from both the MEP and MVA pathways in grapevine. However, the authors do not state whether the overexpressed *VvLinNer* was efficiently taken up by the plastids therefore it is unclear whether the increased nerolidol is due to FPP production within the plastids or that the heterologously expressed VvLinNer may be act within the cytosol. Studies in other plants have indicated the potential for FPP presence in plastids. For example, targeting of FaNES1, a cytosolically localised linalool/nerolidol synthase from strawberry, to plastids in Arabidopsis resulted in an increase in nerolidol abundance, albeit at lower levels than linalool (Aharoni et al., 2004).

Terpene synthases are also able to produce multiple products from a single substrate, a trait that greatly increases terpenoid diversity. Nearly half of the identified mono- and sesquiterpene synthases generate more than one product (Vattekkatte et al., 2018). The ability of TPSs to generate such a wide variety of products is not yet fully understood. One contribution may be the highly reactive carbocationic intermediate that can undergo various reactions to be stabilised. However, single product TPSs do exist, therefore it’s likely that a structural feature of the enzyme contributes to its ability to produce multiple products. No common feature has been identified in TPSs that contribute to their ability to produce multiple products; however, several studies suggest that the conformation of the active site influences this ability (reviewed by Degenhardt et al., 2009; Vattekkatte et al., 2018). For example, the ability of γ-humulene from *A. grandis* to produce 52 different sesquiterpenes was associated with the presence of two DDxxD motifs flanking the active site (Steele et al., 1998; Little & Croteau, 2002). Furthermore, through modelling studies it was shown that TPS4 from *Zea mays* can produce multiple products due to two pockets in the active site which control the conformational change of the carbocationic intermediate (Kollner et al., 2004).

It is important to note that several characterisation studies infer the function of the gene through *in vitro* analysis and heterologous gene expression. A recent study by Salvagnin et al. (2016) highlighted the importance of studying TPS function within its native plant. The authors analysed grapevine *E*-(β)-caryophyllene synthase (*VvGwECar2*) under three conditions: *in vitro*, in a heterologous plant system (Arabidopsis) and in a homologous plant system (*Vitis vinifera).* While the enzyme still produced *E*-(β)-caryophyllene and α-humulene as its major products in all systems, the ratio of these compounds was different in each system. Furthermore, the composition and abundance of secondary products were different in each system. For instance, in the Arabidopsis system thujopsene was also produced, but this was not detected in grapevine.

### 4.4 Functional plasticity of terpene synthases

Another major contribution to terpene diversity is the functional plasticity of TPS active sites, i.e., a single nucleotide substitution can lead to a change in the enzyme function. While TPSs share conserved motifs (e.g., DDxxD and NSE/DTE) that are vital to enzyme function, several structure-function studies have demonstrated that small amino acid substitutions can lead to TPSs producing entirely new products.

Various examples of functional plasticity have been reported for grapevine TPSs. Recent studies into the sesquiterpene rotundone revealed genotypic variation in the cultivars that have high levels of this terpene. Rotundone is responsible for the peppery aroma associated with cultivars such as Shiraz, Cagnulari, Schioppettino, Vespolina, Graciano, and Gruene Veltliner (Mattivi, 2016). A novel allele of the *VvTPS24* gene model, *VvGuaS*, a sesquiterpene synthase whose main product is the rotundone precursor α-guaiene, was identified in Shiraz berries (Drew et al., 2016). Previously, *TPS24* was shown to encode for VvPnSeInt, which produces selina-4,11-diene as its main product (Martin et al., 2010). Drew et al. (2016) also identified two polymorphisms in the *TPS24* gene of Shiraz which is responsible for two non-synonymous amino acid substitutions in the active site of the enzyme resulting in functional conversion of the enzyme from VvPnSeInt to VvGuaS. This is an example of how small genetic variations (single nucleotides) in TPS genes can lead to a complete functional change of the enzymes for which they encode, further increasing the diversity of terpene profiles observed across grapevine cultivars. Additionally, an association study between *VvTer*, an α-terpineol synthase gene, and α-terpineol content in the grape berries derived from 61 cultivars identified two SNPs that associated with higher α-terpineol content. However further study is necessary to ascertain the functional effects of these polymorphisms (Yang et al., 2017a). Another example of the cultivar specific nature of grape TPSs is the recently characterised *(E)-β-farnesene synthase* (*VvMATPS10*) from Muscat of Alexandria flowers (Smit et al., 2019). This gene had been previously characterised to code for a bergamotene synthase (VvGWaBer) in Gewürztraminer (Martin et al., 2010), however when isolated from Muscat of Alexandria it showed a unique sequence and function, producing (*E*)-β-farnesene, as opposed to bergamotene, as a single product. These studies highlight the high functional plasticity of VvTPSs and how this plasticity results in the cultivar-specific functions of VvTPS.

## 5 Secondary modifications of mono- and sesquiterpenes

**Figure 3:**
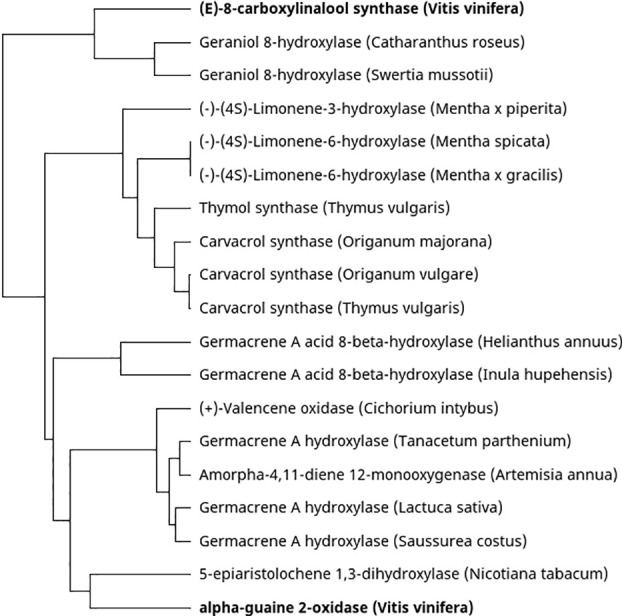
Phylogenetic tree of characterised cytochrome P450 monooxygenases which use mono- or sesquiterpenes as a substrate. The characterised *V. vinifera* CYPs are shown in boldface.

The carbon scaffold of terpenes produced by terpene synthases can be additionally enzymatically modified, further contributing to the diverse terpene profiles of plants. Most terpene modifications are catalysed by cytochrome P450 monooxygenases (CYPs). Regarding grapevine studies, only two CYPs that are involved in terpene modification have been characterised. The first is VvSTO2, which forms the sesquiterpene rotundone by oxygenating its precursor, α-guaiene (Takase et al., 2016b). Secondly, CYP76F14 is a CYP involved in the formation of wine lactone. Wine lactone is a monoterpene which largely contributes to the aroma of Gewürztraminer wines. It is formed during fermentation and aging of wine through a slow, nonenzymatic, acid-catalysed cyclisation from an odourless precursor, (*E*)-8-carboxylinalool. (*E*)-8-carboxylinalool is a grape-derived monoterpene and is synthesised in the berries through the action of CYP76F14 catalysed oxygenation of linalool (Ilc et al., 2017). Congruently, the authors also show that *CYP76F14* maps to a QTL associated with (E)-8-carboxylinalool content in grape berries. **Figure 3** is a phylogenetic analysis comparing functionally characterised CYP genes which act on sesquiterpenes or monoterpenes from other plant species. VvSTO2 (alpha-guaiene oxidase) shows similarity with another CYP which has a bicyclic sesquiterpene substrate from tobacco, 5-epi aristolochene 1,3-hydroxylase. Furthermore, CYP76F14 ((E)-8-carboxylinalool synthase) clusters closely with geraniol 8-hydroxylases from *C. roseus* and *S. mussotii* and linalool and geraniol are both acyclic monoterpene alcohols.

Monoterpenes, and other volatiles, are often present in grapevine as non-volatile glucosides, which are formed through the action of glucosyltransferases. These compounds can either occur as monosaccharides bound to a β-d-glucose moiety or disaccharides with the addition of rhamnose, apiose, or arabinose to the glucose moiety (Hjelmeland & Ebeler, 2015). The formation glucosides are catalysed by glucosyltransferases (GTs). Three GTs, namely VvGT7, VvGT14, and VvGT15, have been functionally characterised in grapevine. All three enzymes accept geraniol, nerol, and citronellol as substrates, with VvGT14 also accepting linalool (Bönisch et al., 2014a; Bönisch et al., 2014b). Additionally, correlation analysis of transcriptomic and metabolic data indicated that UDP-glycosyltransferase 89B2 (LOC100264439) and UDP-glycosyltransferase 83A1 (LOC100248406) potentially contribute to the glycosylation of linalool, hotrienol, α-terpineol, geraniol, and *cis-*rose oxide (Wang et al., 2021).

## 6 Ecophysiological roles of specialised terpenes

**Figure 4:**
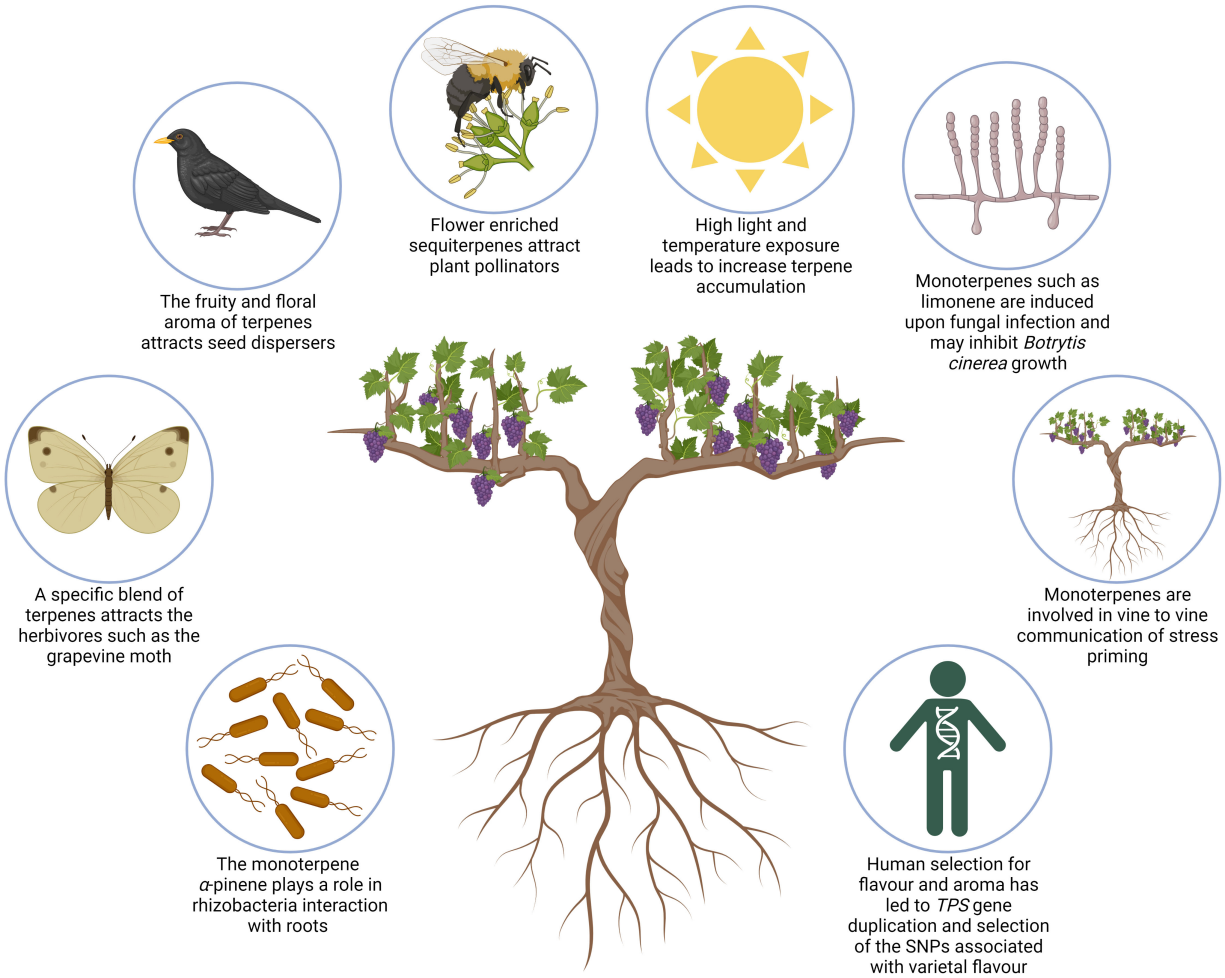
Schematic illustrating ecophysiological roles of mono- and sesquiterpenes in Grapevine. Clockwise, from bottom left, images illustrate the following: monoterpenes have been shown to play a unique function in grapevine roots and their interaction with mutualistic rhizobacteria; the European grapevine moth pest is attracted by a mix of VOCs containing sesquiterpenes; a blend of terpenes is involved in attraction of agents of seed dispersal; sesquiterpenes are the most abundant specialised terpene in grapevine buds and flowers and are involved in attraction of pollinators; light exposure, high temperatures, and UV-B radiation can all increase terpene accumulation in grape berries; several monoterpenes have been found to inhibit fungal growth of *Botrytis cinerea* and *Plasmopara viticola*; monoterpenes such as α-pinene are suggested to play a role in vine-to-tine communication and priming of stress; human selection of cultivars has led to gene duplication of *TPSs* and the selection of SNPs associated with varietal flavour.

The structural diversity of specialised terpenes allows them to fulfil multiple ecological functions related to plant-environment interactions. The following section summarises some of the ecophysiological roles of mono- and sesquiterpenes, particularly as they relate to grapevine (**Figure 4**).

Volatile terpenes attract plant pollinators and seed dispersers (Dudareva et al., 2013). Pollinator attraction has not been associated with an individual compound, but instead a blend of different specialised volatile organic compounds which include terpenes. Furthermore, these compounds may also play a defensive role, protecting the important reproductive organs of the plant against pathogens. Sesquiterpenes are the most abundant specialised terpene in grapevine buds and flowers with sesquiterpene synthase genes showing peak expression in buds and flowers (Matarese et al., 2013; Smit et al., 2019). The precise role of grapevine floral sesquiterpenes in pollinator-attraction or defence is yet to be elucidated. However, domesticated grapevine (*Vitis vinifera*) is hermaphroditic; and self-pollination plays a more dominant role than insect-mediated pollination (Zou et al., 2021). It can therefore be inferred that the role of grapevine flower sesquiterpenes may predominantly be in defence. Alternatively, it is highly likely that human selection for berry and wine aroma is the driving force behind diverse sesquiterpene profiles in domesticated grapevine. This has already been shown for increased monoterpene content associated with *VvDXS1* which underwent a strong selection in Muscats due to human selection during grapevine domestication (Emanuelli et al., 2010).

Individual terpenes or terpene blends that increase its herbivore defence have been identified in different plants (Tholl, 2015; Boncan et al., 2020). The blend and ratio of volatiles emitted as defence is species and herbivore specific. This underlies the role of diverse terpene structure in increasing the overall fitness of individual species for their unique environments. Furthermore, research has shown that some plants release herbivore-induced volatiles which attract the natural predators of herbivores (Tholl, 2015). Regarding grapevine, it has been shown that the European grapevine moth (*Lobesia botrana*) is attracted to a specific blend of (*E*)-β-caryophyllene, (*E*)-4,8-dimethyl-1,3,7-nonatriene (DMNT), and (*E*)-β-farnesene emitted by green grape berries (Tasin et al., 2006). Furthermore, transgenic grapevine lines with modified (*E*)-β-caryophyllene and (*E*)-β-farnesene emissions (three times higher or less than half compared to the wild-type) were shown to effectively interrupt the host-finding ability of grapevine moths (Salvagnin et al., 2018). *Lobesia botrana* is a major pest of vineyards and understanding its host-finding mechanism may lead to the development of sustainable pest treatment strategies aimed at interrupting these mechanisms.

Volatile terpenes are also induced during pathogen infection and have been shown to inhibit pathogen growth (Brilli et al., 2019). *Botrytis cinerea*, a necrotrophic fungus which causes bunchrot in grapevine, has been shown to be inhibited by the monoterpene limonene (Simas et al., 2017). α-pinene, β-pinene, citral, and γ-terpinene were also shown to inhibit *B. cinerea* albeit to a lesser extent. Another major grapevine pathogen, *Plasmopara viticola*, responsible for grapevine downy mildew, could also be inhibited by certain specialised terpenes (Ricciardi et al., 2021). *In vitro* analysis of antifungal activity demonstrated that farnesene, ocimene, nerolidol, and valencene are able to reduce disease severity. The sesquiterpene nerolidol also showed antifungal activity in grapevine, inhibiting the growth of *Phaeoacremonium parasiticum* (Escoriaza et al., 2019).

Plant roots show similar defence responses to the aboveground plant organs. Grapevine roots have been shown to have a distinctive volatile profile when compared with other grapevine vegetative organs in Moscato bianco (Matarese et al., 2014). Myrtenol, borneol, and pinocarveol were more abundant in roots than other organs and are thought to be derived from α-pinene. Furthermore, expression analysis indicated that α-pinene synthase, *VvPNaPin1*, was expressed highest in roots and flower buds. Additionally, α-pinene content increased in grapevine tissues that were inoculated with plant growth promoting rhizobacteria (PGPR), which was isolated from grapevine roots (Salomon et al., 2014; Salomon et al., 2016). These findings may indicate a unique function for α-pinene in grapevine roots and their interaction with mutualistic rhizobacteria.

Additionally, it was previously found that grapevine was able to take up monoterpenes emitted from other plants, such as 1,8-cineole emitted by eucalyptus (*Eucalyptus globulus*) trees (Capone et al., 2012; Pardo-Garcia et al., 2015). This may form part of a plant-to-plant communication systems observed in several species (Rosenkranz et al., 2021). Recent results suggest such communication may occur within and between grapevine plants experience abiotic stress (Midzi et al., 2021). Vines exposed to drought stress appear to be able to prime neighbouring vines through the emission of VOCs such as the monoterpene α-pinene.

Agronomic practices such as leaf removal, training systems and irrigation have traditionally been used to modulate terpene and other volatiles in grapevine to improve the final aroma of the grape berries or wine (Alem et al., 2019). These practices alter the climate around the grapevine and the effect of abiotic factors such as sunlight, water deficit and UV radiation on VOC accumulation, and by extension terpene accumulation, in grapevine has been studied extensively. Furthermore, the influence of climate change on agriculture has also necessitated the understanding of the effect of changing environmental conditions on grapevine quality (Rienth et al., 2021). Lazazzara et al. (2022) provides a comprehensive review of grapevine biogenic VOCs and how they are influenced by various biotic and abiotic factors. Generally, these studies show that light exposure, high temperatures, UV-B radiation and moderate water deficit can all increase terpene accumulation in grape berries indicating that these compounds play a role in the abiotic stress response of plants. In addition to acting as signalling molecules, terpenes, particularly isoprene, are thought to play a role in ROS modulation and membrane stabilisation, however the mechanism of these roles are still poorly understood (Lazazzara et al., 2022).

## 6 Conclusion and future prospects

Grapevine genomic research has made significant contributions to our understanding of specialised terpene metabolism and several key enzymes involved in grapevine monoterpene and sesquiterpene biosynthesis have been identified. The increasing availability of grapevine cultivar genomes have displayed the variation of the *VvTPS* gene family between cultivars and gives insight into *VvTPS* evolution and gene expansion. Furthermore, these studies highlight the limitations of the reference genome with regards to specialised terpene research, as it does not display the cultivar-specific variation of the grapevine TPS family. The availability of more grapevine cultivar genomes and the use of a multi-omics approach may in future provide a more efficient means of identifying and characterising novel *VvTPS*s. Moreover, increased knowledge of grapevine terpene metabolism is agriculturally significant as it can lead to the development of grapevine crops with improved or altered flavour and aroma profiles, while potentially increasing disease resistance.

## Author contributions

RB and JL conceptualised the review and cowrote the manuscript. All authors contributed to the article and approved the submitted version.
